# Supplementary material for: Escherichia coli Increases its ATP Concentration in Weakly Acidic Environments Principally through the Glycolytic Pathway
Source: Genes (Basel). 2020 Aug 25;11(9):991. doi: 10.3390/genes11090991 (PMC7563387; doi:10.3390/genes11090991)
Supplement: Supplementary file 1 [file genes-11-00991-s001.zip › genes-894111-supplementary/Supplement figures/Supplement figures.docx]

Supplement figures


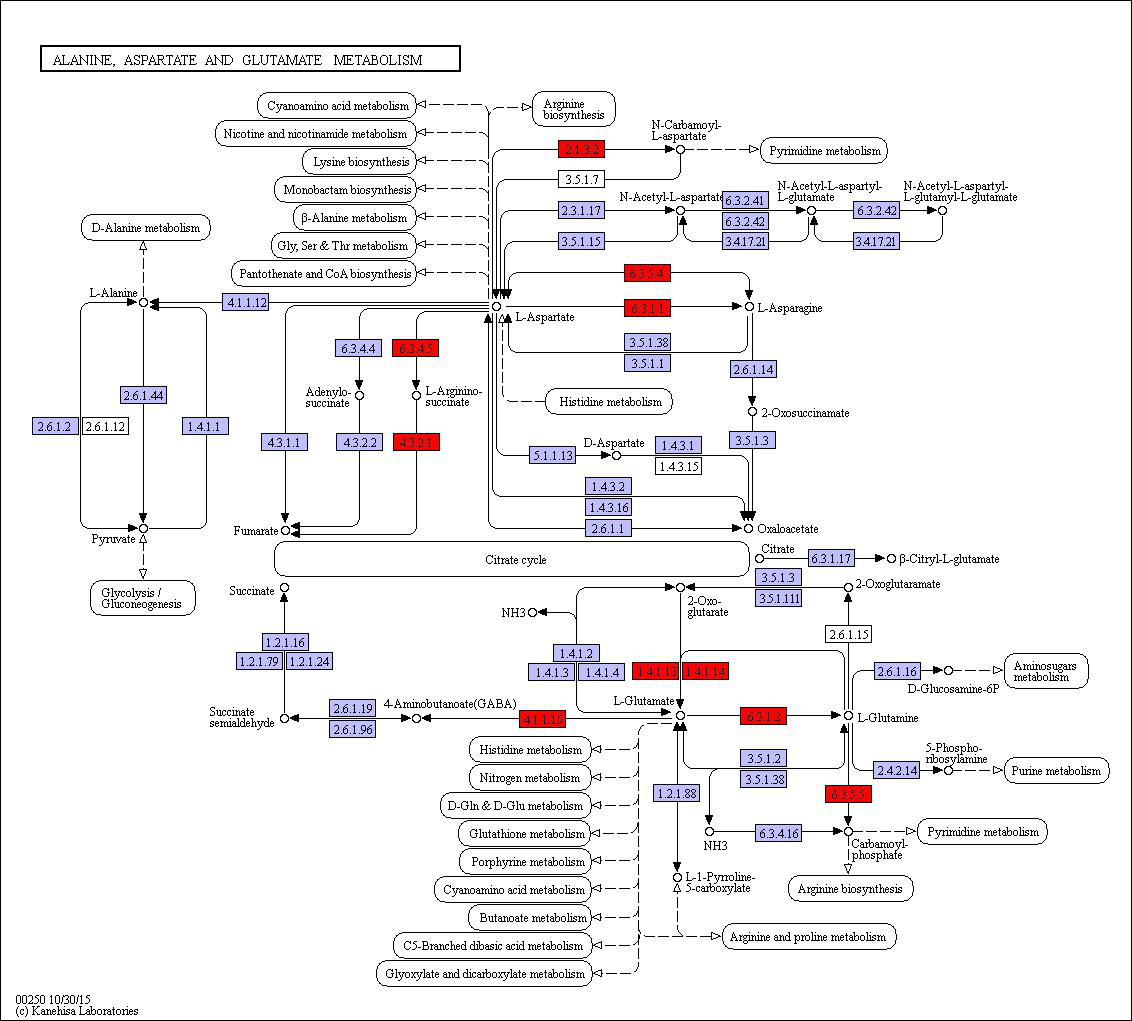


**Figure 1.** The up- and down-regulated genes in amino acid metabolic pathway. The changes in relative expression of *E. coli* amino acid metabolic genes (KEGG pathway) after reducing the pH level of the culture medium from 7.5 to 5.5 revealed the upregulation of genes belonging to particular AR systems. Red genes are up-regulated more than 2 fold, blue genes show no significant change.


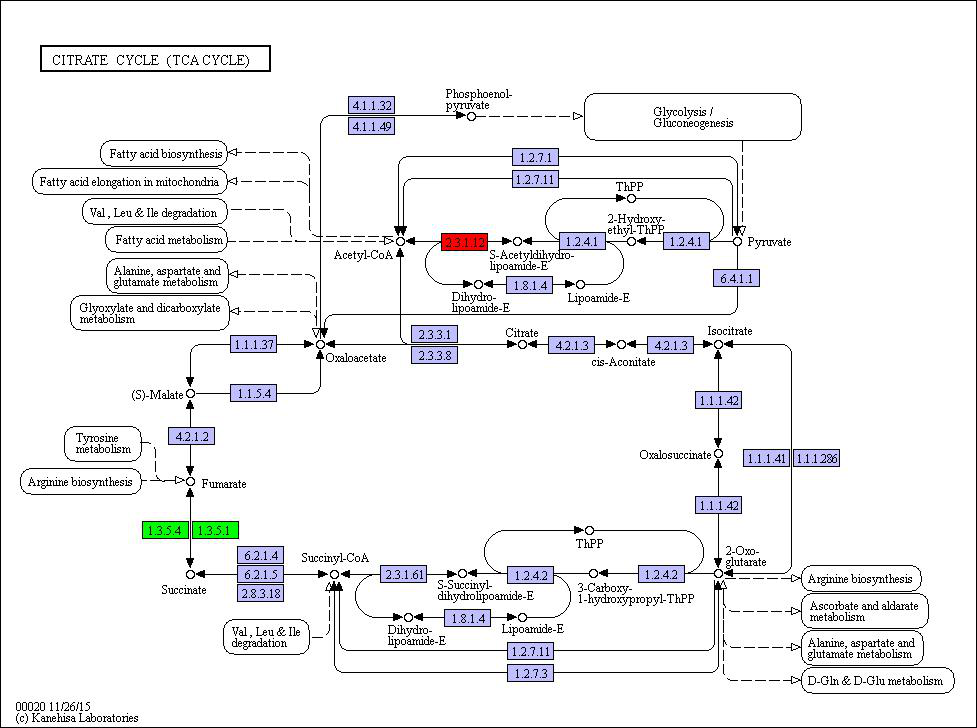


**Figure 2.** The up- and down-regulated genes in the TCA cycle. Red genes are up-regulated more than 2 fold, blue genes show no significant change, and green genes are down-regulated more than 2 fold.
